# Supplementary material for: Subtilase SBT5.2 inactivates flagellin immunogenicity in the plant apoplast
Source: Nat Commun. 2024 Nov 30;15:10431. doi: 10.1038/s41467-024-54790-1 (PMC11608315; doi:10.1038/s41467-024-54790-1)
Supplement: Supplementary file 3 — Description of Additional Supplementary Files [file 41467_2024_54790_MOESM3_ESM.pdf]

Description of Additional Supplementary Files

Supplementary Data 1: Flagellin peptides identified from flagellin incubated in AF of N. benthamiana
Flagellin isolated from *Pta*6605 was incubated with AF of agroinfiltrated *N. benthamiana*. Proteins were precipitated in 80% acetone and the peptides in the supernatant were identified by MS in n=3 replicates.

Supplementary Data 2: Flagellin peptides identified from nonglycosylated flagellin incubated in AF of *N. benthamiana*
Flagellin isolated from the *Δfgt1* mutant of *Pta*6605 was incubated with AF of *N. benthamiana* for 60 minutes. Proteins were precipitated in 80% acetone and the peptides in the supernatant were identified by MS in n=1 replicate.

Supplementary Data 3: Flagellin peptides identified from flagellin incubated with purified SBT5.2-His
Flagellin isolated from *Pta*6605 was incubated with purified SBT5.2-His for 30 minutes. Proteins were precipitated in 80% acetone and the peptides in the supernatant were identified by MS in n=6 replicates.

Supplementary Data 4: Flagellin peptides identified from flagellin incubated in AF of tomato
Flagellin isolated from *Pta*6605 was incubated with AF of tomato for 60 minutes. Proteins were precipitated in 80% acetone and the peptides in the supernatant were identified by MS in n=1 replicate.

Supplementary Data 5: Used parameters for MS experiments.
