# Supplementary material for: Subtilase SBT5.2 inactivates flagellin immunogenicity in the plant apoplast
Source: Nat Commun. 2024 Nov 30;15:10431. doi: 10.1038/s41467-024-54790-1 (PMC11608315; doi:10.1038/s41467-024-54790-1)
Supplement: Supplementary file 8 — Supplementary Data 5 [file 41467_2024_54790_MOESM8_ESM.pdf]

Overview

ACE project	Title
ACE_0383	Comparison of mass spectra among flagellin protein samples
ACE_0481	Hydrolytic release of flagellin peptides.
ACE_0935	Released peptide from flagellin cleavage by SBT5.2a

ACE_0383

File legend

ACE ID	Organism	Organ/ cell line	Treatment/ experimental setup
ACE_0383_PB10	Pseudomonas	flagella	25ul of apoplastic fluids of N. benthamiana transiently expressing P19 mixed with the flagellin proteins isolated from Pseudomonas Pta6605 WT (at 10ng/ul)
ACE_0383_PB11	Pseudomonas	flagella	25ul of apoplastic fluids of N. benthamiana transiently expressing P19 mixed with the flagellin proteins isolated from Pseudomonas Pta6605 WT (at 10ng/ul)
ACE_0383_PB12	Pseudomonas	flagella	25ul of apoplastic fluids of N. benthamiana transiently expressing P19 mixed with the flagellin proteins isolated from Pseudomonas Pta6605 WT (at 10ng/ul)

LC_Settings

MS device	Orbitrap Elite
LC device	Thermo Easy-nLC 1000
ion source	Thermo Nanospray Flex
**Analytical column**	Self-packed fused silica capillary with integrated pico frit emitter; New Objectives PF360-75-15-N-5
column diameter	Length (L_C) = 35 cm; ID = 75µm; OD = 360 µm; emitter 15 µm
stationary phase	Reprosil-Pur 120 C18-AQ, Dr. Maisch GmbH
particle diameter (d_p)	1.9 µm
Pore size	120 Å
Column ID	AC61
Column oven	Sonation column oven PRSO-V1
Column oven temp.	45°C
**solvents**	A: 0.1% FA in UPLC water<br>B: 0.1% FA in 80% UPLC ACN
gradient	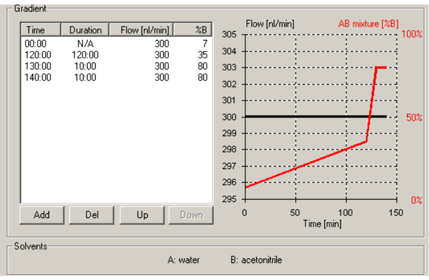

MS_Settings

Project	MS	general	MS1	MS2	MS2	MS3	Comments; special settings
ACE_0383	Elite	Tune v2.7.0.1112 SP2<br>Xcalibur: v3.0.63<br>Gradient: 140 min	Analyzer: FT<br>Res.: 60000<br>SR: 300 - 1700<br>AGC: 3×10^6<br>AcT: 50 ms<br>RF: n/a<br>SF: --<br>DDM: NS/15	Analyzer: IT<br>Res./ScR: -/rapid<br>SR: Auto<br>AGC: 1×10^4<br>AcT: 50 ms<br>CS: > +2<br>IsM: IT<br>IsW: 2.0<br>Frag.: CID<br>NCE: 35			classic orbitrap experiment: MS1 in Orbitrap at high resolution and data dependent MS2 in Iontrap at rapid scan rate. Dynamic exclusion enabled (exclude after n times=1; Exclusion duration (s)= 120; mass tolerance= ± 10 ppm)<br><br>capillary temp.: 200 °C

Note: **FT**= Fourier Transform (Orbitrap); **IT**= Iontrap; **Q**= Quadrupol; **Res.**= max. Resolution at 200 m/z (Lumos) or 400 m/z (Elite) [FWHM (full width at half maximum)]; **ScR**= scan rate for measurements in the IT; **SR**= scan range [m/z]; **AGC**= automatic gain control, max number of acquired ions per measurement; **AcT**= max. Ion acquisition time [ms]; **CS**= charge states used for fragmentation; **IsM**= Isolation mode (Q or IT), MS2 isolation and further is only done in IT; **IsW**= Isolation window [m/z], value followed by scan mode the isolation is based on (MS1, MS2 …) **Frag.**= Fragmentation method; **HCD**= Higher-

energy collisional dissociation; **CID**= Collision-induced dissociation; **ETD**= Electron-transfer dissociation**; EThcD=** Electron-Transfer/Higher-Energy Collision Dissociation; **sHCD**= stepped HCD**; NCE**= normalized collision energy; **cycles**: number of MSn recorded or max cycle time; RF= RF Lens [%]; **SF**= Source Fragmentation [V]; **DDM**: Data dependent Mode (cycle time in seconds, CT/[s] or number of scans, NS); **NS**= Number of data dependent scans

Search Settings

Program & version	MaxQuant v1.5.5.30.
Search engine	Andromeda
settings	Basically default; LFQ and MBR were turned on
Static modification	none
Digestion mode	unspecific
Dynamic modification	Acetyl (N-term); Oxidation (M)
Modification included in quantification	Oxidation (M)
Databases	1. Contaminants<br>2. p19_vector_proteins.fasta<br>3. ACE_0383_SOI_v02.fasta

ACE_0481

File legend

ACE ID	Organism	Organ/ cell line	Treatment/ experimental setup
ACE_0481_PB01	*Pseudomonas*	flagella	25 µl of AF from *N. benthamiana* overexpressing P19+**EV** and mixed with the flagellin proteins isolated from Pseudomonas ***Pta*6605**Δ***fgt1*** (at 10ng/µl)
ACE_0481_PB05	*Pseudomonas*	flagella	25 µl of AF from Tomato incubated with **DMSO** and mixed with the flagellin proteins isolated from Pseudomonas ***Pta*6605** (at 10ng/µl)

LC_Settings

MS device	Thermo Orbitrap Fusion Lumos
LC device	Thermo Easy-nLC 1200
ion source	Thermo Nanospray Flex
**Analytical column**	Self-packed fused silica capillary with integrated pico frit emitter; New Objectives PF360-75-15-N-5
column diameter	Length (L_C) = 30 cm; ID = 75µm; OD = 360 µm; emitter 15 µm
stationary phase	Reprosil-Pur 120 C18-AQ, Dr. Maisch GmbH
particle diameter (d_p)	1.9 µm
Pore size	120 Å
Column ID	Ac82
Column oven	Sonation column oven PRSO-V2
Column oven temp.	50°C
**solvents**	A: 0.1% FA in UPLC water<br>B: 0.1% FA in 80% UPLC ACN
gradient	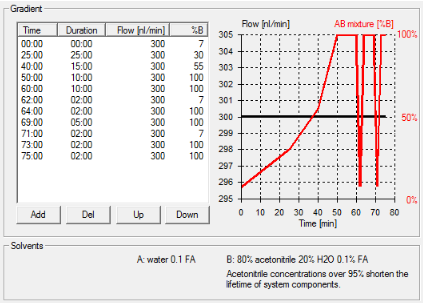

MS_Settings

Project	MS	general	MS1	MS2	MS2	MS3	Comments; special settings
ACE_0481	Lumos	Tune v3.5.3881.18<br>Xcalibur v4.5.445.18<br>Gradient: 75 min	Analyzer: FT<br>Res.: 120000<br>SR: 375 - 1500<br>AGC: Standard<br>AGC abs.: 400000<br>AcT: 50 ms<br>RF: 30<br>SF: --<br>DDM: CT/3sec	Analyzer: IT<br>Res./ScR: -/rapid<br>SR: Auto<br>AGC: 100%<br>AGC abs.: 10000<br>AcT: 35 ms<br>CS: +2 to +7<br>IsM: Q<br>IsW: 1.2<br>Frag.: CID<br>NCE: 35			classic orbitrap experiment: MS1 in Orbitrap at high resolution and data dependent MS2 in iontrap. Dynamic exclusion enabled (exclude after n times=1; Exclusion duration (s)= 60; mass tolerance= ± 10ppm)<br><br>Intensity Threshold: 5000<br>Ion transfer Tube Temp: 275 °C<br>Ion Source Voltage: 2500 V

Note: **FT**= Fourier Transform (Orbitrap); **IT**= Iontrap; **Q**= Quadrupol; **Res.**= max. Resolution at 200 m/z (Lumos) or 400 m/z (Elite) [FWHM (full width at half maximum)]; **ScR**= scan rate for measurements in the IT; **SR**= scan range [m/z]; **AGC**= automatic gain control, max number of acquired ions per measurement; **AcT**= max. Ion acquisition time [ms]; **CS**= charge states used for fragmentation; **IsM**= Isolation mode (Q or IT), MS2 isolation and further is only done in IT; **IsW**= Isolation window

[m/z], value followed by scan mode the isolation is based on (MS1, MS2 …) **Frag.**= Fragmentation method; **HCD**= Higher-energy collisional dissociation; **CID**= Collision-induced dissociation; **ETD**= Electron-transfer dissociation**; EThcD=** Electron-Transfer/Higher-Energy Collision Dissociation; **sHCD**= stepped HCD**; NCE**= normalized collision energy; **cycles**: number of MSn recorded or max cycle time; RF= RF Lens [%]; **SF**= Source Fragmentation [V]; **DDM**: Data dependent Mode (cycle time in seconds, CT/[s] or number of scans, NS); **NS**= Number of data dependent scans

Search Setting

Program & version	MaxQuant v1.6.3.4
Search engine	Andromeda
settings	Basically default; LFQ and MBR were turned on
Static modification	none
Digestion mode	unspecific
Dynamic modification	Acetyl (N-term); Oxidation (M)
Modification included in quantification	Oxidation (M)
Databases	1. Contaminants<br>2. ACE_0481_SOI_v02.fasta<br>3. p19_vector_proteins.fasta<br>4. 12864_2019_6058_MOESM10_ESM.fasta (*N. benthamiana*))<br>5. UP000000813_176299.fasta (*A. tumefaciens strain C58*)

ACE_0935

File legend

ACE ID	Organism	Organ/ cell line	Treatment/ experimental setup
ACE_0935_PB04	*N. benth;P. syringae*		Flagellin +GFP 30 min - replicate 1
ACE_0935_PB05	*N. benth;P. syringae*		Flagellin +GFP 30 min - replicate 2
ACE_0935_PB06	*N. benth;P. syringae*		Flagellin +GFP 30 min - replicate 3
ACE_0935_PB13	*N. benth;P. syringae*		Flagellin +SBT5.2a 30 min - replicate 1
ACE_0935_PB14	*N. benth;P. syringae*		Flagellin +SBT5.2a 30 min - replicate 2
ACE_0935_PB15	*N. benth;P. syringae*		Flagellin +SBT5.2a 30 min - replicate 3

Note: HIS-tagged GFP and SBT5.2a were expressed in N. benthamiana leaves and purified from leaves AF. Native flagellin proteins were isolated from Pseudomonas syringae pv. tabaci strain 6605.

LC_Settings

MS device	Orbitrap Fusion Lumos
LC device	Thermo Vanquish Neo
ion source	Thermo Nanospray Flex
**Analytical column**	Self-packed fused silica capillary with an integrated sintered frit; CoAnn Technologies ICT36007515F-50-5
column diameter	Length (L_C) = 28 cm; ID = 75µm; OD = 360 µm; emitter 15 µm
stationary phase	Phenomenex Kinetex C18-XB core shell
particle diameter (d_p)	1.7 µm
Pore size	120 Å
Column ID	AC157/AC158
Column oven	Sonation column oven PRSO-V2
Column oven temp.	50°C
**solvents**	A: 0.2% FA, 2% ACN, 98% H_2O<br>B: 0.2% FA, 80% ACN, 20 % H_2O
gradient	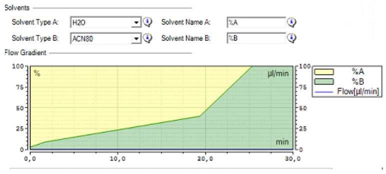

No	Time	Duration [min]	Flow [µl/min]	%B	Volume [µl]	No. of Column Volumes
1	0,000	Run				
2	0,000	0,000	0,250	3,0	0,00	0,00
3	1,660	1,660	0,250	9,0	0,42	0,50
4	19,340	17,680	0,250	40,0	4,42	5,33
5	25,340	6,000	0,250	100,0	1,50	1,81
6	26,000	0,660	0,250	100,0	0,17	0,20
7	26,000	Column Wash				
8	30,000	4,000	0,250	100,0	1,00	1,21
9	30,000	Stop Run				
10	30,000	Column Equilibration				

MS_Settings

Project	MS	general	MS1	MS2	MS2	MS3	Comments; special settings
ACE_0935	Lumos	Tune v4.1.4244<br>Xcalibur v4.7.69.37<br>SII: 1.7.0.468<br>Gradient: 30 min	Analyzer: FT<br>Res.: 120000<br>SR: 375 - 1500<br>AGC: Standard<br>AGC abs.: 400000<br>AcT: 50 ms<br>RF: 30<br>SF: --<br>DDM: CT/3sec	Analyzer: IT<br>Res./ScR: -/rapid<br>SR: Auto<br>AGC: 300%<br>AGC abs.: 30000<br>AcT: 35 ms<br>CS: +2 to +7<br>IsM: Q<br>IsW: 1.6<br>Frag.: HCD<br>NCE: 32			classic orbitrap experiment: MS1 in Orbitrap at high resolution and data dependent MS2 also in Orbitrap high resolution. Dynamic exclusion enabled (exclude after n times=1; Exclusion duration (s)= 20; mass tolerance= ± 10ppm)<br>Intensity Threshold: 2000<br>Ion transfer Tube Temp: 250 °C<br>Ion Source Voltage: 2500 V

Note: **FT**= Fourier Transform (Orbitrap); **IT**= Iontrap; **Q**= Quadrupol; **Res.**= max. Resolution at 200 m/z (Lumos) or 400 m/z (Elite) [FWHM (full width at half maximum)]; **ScR**= scan rate for measurements in the IT; **SR**= scan range [m/z]; **AGC**= automatic gain control, max number of acquired ions per measurement; **AcT**= max. Ion acquisition time [ms]; **CS**= charge states used for fragmentation; **IsM**= Isolation mode (Q or IT), MS2 isolation and further is only done in IT; **IsW**= Isolation window

[m/z], value followed by scan mode the isolation is based on (MS1, MS2 …) **Frag.**= Fragmentation method; **HCD**= Higher-energy collisional dissociation; **CID**= Collision-induced dissociation; **ETD**= Electron-transfer dissociation**; EThcD=** Electron-Transfer/Higher-Energy Collision Dissociation; **sHCD**= stepped HCD**; NCE**= normalized collision energy; **cycles**: number of MSn recorded or max cycle time; RF= RF Lens [%]; **SF**= Source Fragmentation [V]; **DDM**: Data dependent Mode (cycle time in seconds, CT/[s] or number of scans, NS); **NS**= Number of data dependent scans

Search Settings

Program & version	MaxQuant v2.5.2.0
Search engine	Andromeda
settings	Basically default; LFQ and MBR were turned on
Static modification	none
Digestion mode	unspecific
	Min. peptide length for unspecific search 7<br>Max. peptide length for unspecific search 36
Dynamic modification	Acetyl (N-term); Oxidation (M)
Modification included in quantification	Oxidation (M);Acetyl (Protein N-term)
Databases	1. Contaminants<br>2. ACE_0935_SOI_v01.fasta
